# When Is a Sprint a Sprint? A Review of the Analysis of Team-Sport Athlete Activity Profile

**DOI:** 10.3389/fphys.2017.00432

**Published:** 2017-06-20

**Authors:** Alice J. Sweeting, Stuart J. Cormack, Stuart Morgan, Robert J. Aughey

**Affiliations:** ^1^Institute of Sport, Exercise and Active Living (ISEAL), Victoria UniversityFootscray, VIC, Australia; ^2^Netball AustraliaFitzroy, VIC, Australia; ^3^Performance Research, Australian Institute of SportBruce, ACT, Australia; ^4^School of Exercise Science, Australian Catholic UniversityFitzroy, VIC, Australia; ^5^Department of Rehabilitation, Nutrition and Sport, School of Allied Health, La Trobe UniversityBundoora, VIC, Australia

**Keywords:** velocity thresholds, acceleration, data mining, player tracking, match analysis

## Abstract

The external load of a team-sport athlete can be measured by tracking technologies, including global positioning systems (GPS), local positioning systems (LPS), and vision-based systems. These technologies allow for the calculation of displacement, velocity and acceleration during a match or training session. The accurate quantification of these variables is critical so that meaningful changes in team-sport athlete external load can be detected. High-velocity running, including sprinting, may be important for specific team-sport match activities, including evading an opponent or creating a shot on goal. Maximal accelerations are energetically demanding and frequently occur from a low velocity during team-sport matches. Despite extensive research, conjecture exists regarding the thresholds by which to classify the high velocity and acceleration activity of a team-sport athlete. There is currently no consensus on the definition of a sprint or acceleration effort, even within a single sport. The aim of this narrative review was to examine the varying velocity and acceleration thresholds reported in athlete activity profiling. The purposes of this review were therefore to (1) identify the various thresholds used to classify high-velocity or -intensity running plus accelerations; (2) examine the impact of individualized thresholds on reported team-sport activity profile; (3) evaluate the use of thresholds for court-based team-sports and; (4) discuss potential areas for future research. The presentation of velocity thresholds as a single value, with equivocal qualitative descriptors, is confusing when data lies between two thresholds. In Australian football, sprint efforts have been defined as activity >4.00 or >4.17 m·s^−1^. Acceleration thresholds differ across the literature, with >1.11, 2.78, 3.00, and 4.00 m·s^−2^ utilized across a number of sports. It is difficult to compare literature on field-based sports due to inconsistencies in velocity and acceleration thresholds, even within a single sport. Velocity and acceleration thresholds have been determined from physical capacity tests. Limited research exists on the classification of velocity and acceleration data by female team-sport athletes. Alternatively, data mining techniques may be used to report team-sport athlete external load, without the requirement of arbitrary or physiologically defined thresholds.

## Introduction

The quantification of athlete external load is of interest to scientists and practitioners, for the planning and monitoring of training or competition. Team-sport athlete external load can be quantified using accelerometers, global positioning systems (GPS), local positioning systems (LPS), and optical tracking systems. Except for accelerometers, these systems calculate displacement, velocity and acceleration over time. The analysis of external load over a match or training session is termed activity profile (Aughey, 2011a). Information from the activity profile is used to monitor change across a competitive season or tournament (Bradley et al., 2009; Jennings, D. et al., 2012) and allow for the design of specific training drills (Boyd et al., 2013).

The activity profile of field-based team-sport athletes is well-documented (Aughey, 2011a; Mooney et al., 2011; Jennings, D. H. et al., 2012; Bradley et al., 2013). Activity profile analysis typically includes time spent in velocity or acceleration zones. These zones are defined according to threshold values and determined arbitarily, by the proprietary software of tracking systems or expressed relative to a physiological test. Currently, there is no consensus on how to determine a velocity or acceleration threshold. Large discrepancies exist in the classification of a sprint effort. The comparison of activity profiles across and within team-sports is consequently difficult.

The aim of this narrative review is to examine the varying velocity and acceleration thresholds used to analyze team-sport athlete external load. Applying a global velocity or acceleration threshold does not account for individual differences. Whilst thresholds can be individualized, physiological tests comprising continuous or linear movement do not reflect changes of direction and acceleration. The current techniques used to analyze external load are therefore inappropriate. Alternate methods, including unsupervised data mining techniques, are considered. These techniques find trends within external data and may be useful in informing thresholds.

## Athlete tracking technologies

Team-sport athlete external load is collected by tracking technologies. Manual video analysis is an inexpensive method to estimate external load. Athletes are filmed by cameras positioned around a playing area, with footage subjectively coded into locomotor categories (Spencer et al., 2004). Manual video analysis requires substantial time demand to examine activity. Validity also has not been established, due to the subjective estimation of athlete movement. A tracking system must be valid so meaningful changes in athlete activity profile can be detected. The capacity of a human to consistently reproduce results is also a major limitation of manual video analysis. Semi-automated tracking systems were designed to remove the laborious and subjective classification of athlete activity. Commercial systems, including ProZone (Di Salvo et al., 2006) and Amisco (Castellano et al., 2014), can detect the position of multiple team-sport athletes. However, the required equipment is expensive and non-portable. Activity profiles therefore cannot be collected without the elaborate infrastructure. Athlete movement is also collected in a two-dimensional plane, with changes in position due to vertical movement going undetected (Barris and Button, 2008).

Accelerometers are wearable sensors that directly quantify athlete load in three-dimensional planes. Accelerometers have been utilized in field-based (Mooney et al., 2013) and court-based (Cormack et al., 2014) team-sports however, accelerometers cannot calculate an athlete's position relative to a playing area. Consequently, the time and distance covered by an athlete at varying velocities are unable to be quantified. The use of GPS to collect the distance and velocities of field-based team-sport athletes is well-documented (Buchheit et al., 2010b; Jennings, D. H. et al., 2012; Varley et al., 2013b). A recent review has examined factors influencing the setup, analysis and reporting of GPS data, for use in team-sports (Malone et al., 2016).

Large variations exist in GPS estimates of changes in velocity, between models and units from the same manufacturer (Buchheit et al., 2014). During simultaneous capture of a sled dragging exercise, small to very large between-model and unit differences were observed in 15 Hz GPS units (Buchheit et al., 2014). These units were manufactured with a 10 Hz GPS but upsampled to 15 Hz (Aughey, 2011a). In 10 Hz GPS, acceleration and deceleration movements have a large between-unit coefficient of variation (CV) of 31–56% (Varley et al., 2012). A variety of factors may influence GPS measures of acceleration and velocity. The accuracy of GPS to measure instantaneous velocity is limited by unit processing speed, location, antenna volume, and chipset capacity. Quantification of instantaneous velocity is up to three times more accurate in 10 Hz GPS units compared to 5 Hz (Varley et al., 2012). When measuring acceleration and deceleration, 10 Hz units still differ by ~10% when compared to a laser device (Varley et al., 2012).

Whilst GPS quantifies the position and velocities of field-based team-sport athletes (Aughey, 2011a), GPS cannot be used with court-based sports held indoors, due to no satellite reception. The development of radio-frequency (RF) based LPS, including the Wireless ad hoc System for Positioning (WASP), allows athlete movement to be captured indoors (Hedley et al., 2010). Local position systems (LPS) sample at up to 1000 Hz with generally superior accuracy compared to GPS (Stevens et al., 2014). During varying speed and change of direction movement, the average acceleration and deceleration derived from LPS was within 2% of Vicon (Stevens et al., 2014). Although, accuracy for peak acceleration and deceleration is limited, LPS can measure average change in velocity or time spent in various acceleration thresholds.

## Distance covered

A common athlete activity profile measure is the total distance covered. English Premier League athletes cover an average of 10,714 m during matches (Bradley et al., 2009), less than One Day International (ODI) cricketers at 15,903 m per match (Petersen et al., 2009). Elite Australian footballers may record total distances of up to 12,939 m (Coutts et al., 2010). The total distance covered during matches varies across athlete age (Buchheit et al., 2010a), position and competition level (Jennings, D. H. et al., 2012). When total distance covered is expressed per minute of match duration, soccer athletes cover 104 m·min^−1^ (Varley et al., 2013b). Australian footballers may average 157 m·min^−1^ (Aughey, 2011b) whilst elite rugby league players cover up to 97 m·min^−1^ (Varley et al., 2013b). Sport-specific constraints, including positional or tactical roles, may contribute to these differences. The higher total distance in Australian football may be attributed to the unlimited interchange policy (removed in 2015), and the smaller field size available to soccer and rugby league athletes (Varley et al., 2013b). The total distance covered should be presented per minute of match duration or time spent on field/ in a training drill (Aughey, 2011a).

Court-based athletes have a smaller playing area compared to their field-based counterparts, yet cover similar meters per minute. There is limited activity profile research on court-based athletes. State-level female basket ballers cover 127–136 m·min^−1^ during matches (Scanlan et al., 2012), higher than junior males (115 m·min^−1^) and similar to state- (126–132 m·min^−1^) and national (130–133 m·min^−1^) male basketballers (Scanlan et al., 2011). In semi-elite netball, center (C) athletes cover up to 133 m·min^−1^ compared to goal keepers (GK) and goal shooters (GS), who average 71 and 70 m·min^−1^, respectively (Davidson and Trewartha, 2008). These differences could be due to the spatial restrictions imposed by each playing position although manually estimating distance covered from video may also provide unreliable estimates (Barris and Button, 2008).

In court-based sports, the ball may frequently and chaotically change direction. Court-based athletes must be responsive to movement of the ball, their team-mates and opposition in a small area. Athletes may change direction and complete short, high-intensity movements to cover or create space. Although, there are more spatial limitations compared to field-based sports, the high frequency of these actions performed by court-based athletes may result in a comparable meters per minute profile. Whilst reporting meters per minute gives an understanding of intensity, granular periods of activity at different velocities are lost by aggregating to the total distance covered. Quantifying the time spent and distance covered at varying velocities may be useful in programming training and monitoring load.

## Velocity thresholds

During matches or training, the instantaneous velocity of an athlete is binned into different zones via threshold values. Velocity thresholds are defined by proprietary software providers (Cunniffe et al., 2009), modified from published research (Jennings, D. H. et al., 2012) or determined arbitrarily (Mohr et al., 2003). There is no consensus on how to determine a velocity threshold and large discrepancies exist, even within a single team-sport (Table 1). The comparison of activity profile research is consequently difficult.

**Table 1:** Classification of athlete movement, according to speed zones, in a variety of field-based team-sports.

**Reference**	**Cohort**	**Zone 1**		**Zone 2**		**Zone 3**		**Zone 4**		**Zone 5**		**Zone 6**	
		**Speed (m·s^−1^)**	**Descriptor**	**Speed (m·s^−1^)**	**Descriptor**	**Speed (m·s^−1^)**	**Descriptor**	**Speed (m·s^−1^)**	**Descriptor**	**Speed (m·s^−1^)**	**Descriptor**	**Speed (m·s^−1^)**	**Descriptor**
**RUGBY UNION**													
Clarke et al., 2014	Elite females	<2	Low	>3.5	Moderate					>5	High-intensity		
Suárez-Arrones et al., 2012	Elite males	0.03–1.64	Standing and walking	1.66–3.31	Jogging	3.33–3.86	Cruising	3.89–4.98	Striding	5–5.53	High-intensity	>5.56	Sprinting
**COMBINED—SOCCER, RUGBY LEAGUE, AND AUSTRALIAN FOOTBALL**													
Varley et al., 2013b	Elite males	0–5.4	Low-intensity							≥5.5–10	High-velocity	≥7–10	Sprinting
**AUSTRALIAN RULES FOOTBALL**													
Sullivan et al., 2013	Elite males									>4	High-speed		
Aughey, 2010	Elite males	0.10–4.17	Low-intensity							4.17–10	High-intensity		
**Hockey**													
Jennings, D. et al., 2012	Elite males	0.10–4.17	Low-speed							>4.17	High-speed		
Macutkiewicz and Sunderland, 2011	Elite females	0–0.17	Standing	0.19–1.67	Walking	1.69–3.06	Jogging	3.08 to 4.17	Running	4.19–5.28	Fast-running	>5.28	Sprinting
**RUGBY LEAGUE**													
Johnston et al., 2013	Sub-elite males	0–4.72	Low-speed							>4.75	High-speed		
Kempton et al., 2015a	Elite males							>4	High-speed	>5.03	Very high-speed	>6.67	Sprinting
**SOCCER**													
Buchheit et al., 2010a	Youth males	<3.61	Low-intensity					3.64–4.44	High-intensity	4.47–5.28	Very high	>5.31	Sprinting
Carling et al., 2012	Elite males	<0.17	Standing	1.94–1.97	Walking	2–3.97	Jogging	4–5.47	Running	>5.5	High-intensity		

The inconsistency between velocity thresholds extends to qualitative descriptors. For example, activity may be labeled as low-velocity or low-intensity movement. Low-velocity movement, including walking and jogging, could be activity between 0 and up to 5.40 m·s^−1^ (Varley et al., 2013b). Yet in the same sport, activity >4.00 m·s^−1^ was classed as high-speed running (Sullivan et al., 2013). The classification of high-velocity or high-intensity movement is also without consistent definition. The varying definitions make for a difficult comparison between studies. In Australian football, sprint efforts have been defined as activity >4.00 m·s^−1^ (Sullivan et al., 2013) while a threshold of >4.17 m·s^−1^ has also been utilized (Aughey, 2010; Mooney et al., 2011). The presentation of thresholds as a single > or < value, with ambiguous descriptors, is confusing when velocity data falls between two thresholds. For example, running by professional soccer athletes is described as velocities between 4.00 and 5.47 m·s^−1^ whilst activity >5.50 m·s^−1^ was considered high-intensity movement (Carling et al., 2012). It is unclear if velocities within the 0.03 m·s^−1^ upper and lower ranges of the two classifications were removed from analysis. Deletion of these values may influence the frequencies and durations reported. Research describing thresholds in this manner should detail how instantaneous velocities are binned into different zones. If researchers use discrete values, it is recommended that thresholds be presented as ≥ or ≤ values.

The confusion in velocity thresholds also extends to the duration of a sprint. In elite female rugby union (Clarke et al., 2014), hockey (Vescovi, 2014), and professional male soccer (Carling et al., 2012) matches, sprinting must occur for a minimum of 1 s. However, in other studies (Buchheit et al., 2010a; Jennings, D. H. et al., 2012; Varley et al., 2013b; Kempton et al., 2015b), the minimum duration is not stated. It is unclear what effect these inconsistent minimum threshold durations have on the activity profile. Researchers should state the minimum duration required to record a sprint effort. The inconsistency of sprint thresholds in the literature is likely due to values being arbitrarily determined or taken from proprietary software.

## Acceleration thresholds

Acceleration is a metabolically demanding activity, requiring more energy than constant running (Osgnach et al., 2010). During team-sport matches, a large number of high intensity efforts are short in duration and commence from a low velocity. In elite soccer matches, more than 85% of maximal accelerations did not exceed the high-speed (4.17 m·s^−1^) threshold (Varley and Aughey, 2013). Maximal accelerations (>2.78 m·s^−2^) occurred eight times more than sprinting, classified as >6.94 m·s^−1^ but <10.00 m·s^−1^ (Varley and Aughey, 2013). The starting velocity is critical when measuring accelerations or decelerations, although quantification of these variables is dependent upon the validity and reliability of athlete tracking systems.

There are large inconsistencies between acceleration thresholds used throughout the literature. In field-based team-sports, accelerations have been classified as >1.11 m·s^−2^ (Wisbey et al., 2010), 2.78 m·s^−2^ (Varley et al., 2013a), 3.00 m·s^−2^ (Hodgson et al., 2014), and 4.00 m·s^−2^ (Farrow et al., 2008). Accelerations have also been categorized into moderate (2.00–4.00 m·s^−2^) or high (>4.00 m·s^−2^) zones, with a minimum duration of 0.40 s (Higham et al., 2012). The rationale used to select these zones is unknown. The 2.78 m·s^−2^ threshold used in soccer (Varley and Aughey, 2013) and Australian Football (Aughey, 2010) originated from a standing start maximal acceleration of between 2.50 and 2.70 m·s^−2^, performed by non-athletes (Varley et al., 2012). Since elite Australian Football athletes often maximally accelerate from a moving start during matches (Aughey and Falloon, 2008), a 4.00 m·s^−2^ threshold was considered too high and 1.11 m·s^−2^ too low (Aughey, 2010). It appears the threshold of 2.78 m·s^−2^ was determined arbitrarily (Aughey, 2010). Acceleration thresholds of 1.50, 3.00, and 4.00 m·s^−2^ have been used in a single study (Buchheit et al., 2014). Specifying thresholds in this manner has implications for quantifying activity profile and monitoring change over time, particularly when large variations in the measurement of acceleration are common between GPS models from the same manufacturer (Buchheit et al., 2014).

The velocity distribution of elite field-based team-sport athletes was used to create sport-specific threshold values (Dwyer and Gabbett, 2012). Match data from five elite female and male soccer, hockey and professional male Australian Football athletes were collected from GPS sampling at 1 Hz (Dwyer and Gabbett, 2012). A frequency distribution of speed (0–7 m·s^−1^) in 0.1 m·s^−1^ increments was computed from the 25 data sets and an average distribution calculated (Dwyer and Gabbett, 2012). Four normally distributed Gaussian curves were then fitted to the averaged velocity distribution curves and the intersecting points used to determine thresholds for each sport (Dwyer and Gabbett, 2012). A frequency distribution of acceleration from each data set was calculated and a threshold was based on the highest 5% of accelerations performed (Dwyer and Gabbett, 2012). This threshold was then calculated for each pre-determined velocity range and used to identify sprints (Dwyer and Gabbett, 2012). The average velocity distribution for all field-based team-sports was similar. Differences between sexes from the same sport were larger than differences across sports (Dwyer and Gabbett, 2012). Six additional sprints, of a short duration, would not have been recorded using the traditional threshold (Dwyer and Gabbett, 2012). While the decision to include five movement categories comprising standing, walking, jogging, running, and sprinting, appear to have been arbitrarily determined, this is a novel idea compared to the traditional analysis of athlete velocity. This approach was utilized to profile the activity of national level lacrosse (Polley et al., 2015) and youth female field hockey (Vescovi, 2014) athletes. However, the 1 Hz GPS units used have a very large (77.2%) CV when measuring short sprint efforts (Jennings et al., 2010). Consequently, data obtained from 1 Hz GPS during these movements, and the results presented, should be interpreted with extreme caution. The small sample size is also limited in detecting meaningful change across and between sports. Decelerations or negative changes in velocity were also removed from the analysis, likely due to the poor capacity of GPS to accurately quantify these movements (Buchheit et al., 2014).

The ability to reduce velocity is termed deceleration. An athlete's capacity to efficiently decelerate is important for changing direction (Kovacs et al., 2008). The major components of deceleration include dynamic balance, power, reactive, and eccentric strength (Kovacs et al., 2008). In elite team-sport athletes, the substantial eccentric loading during repeated decelerations is likely to have a detrimental effect on subsequent 40 m sprint test performance (Lakomy and Haydon, 2004). In collegiate team-sport athletes, muscle damage was induced post 15 × 30 m repeated sprints with a rapid deceleration, interspersed with 60 s of passive recovery (Howatson and Milak, 2009). Increased muscle soreness, swelling, creatine kinase efflux and decreased maximum isometric contract was also observed 48–72 h post exercise (Howatson and Milak, 2009). Collectively, these results demonstrate the magnitude of muscle and performance damage when team-sport athletes perform repeated deceleration efforts.

Investigation into the decelerations of team-sport athletes during matches is limited. In elite male rugby seven matches, decelerations were classified as moderate (−4.00 to −2.00 m·s^−2^) or high (> 4.00 m·s^−2^) and occurred for a minimum of 0.40 s (Higham et al., 2012). It is unclear why these zones were chosen. A 35 and 25% difference in moderate and high decelerations, respectively, existed between standards of play (Higham et al., 2012). The large error of 5 Hz GPS to accurately quantify these movements may account for the difference between playing levels. The deceleration of professional rugby league athletes were investigated during two competitive seasons (Delaney et al., 2015). Differences in the maximum value recorded over a rolling average, from 1 to 10 min in duration, was compared across playing positions (Delaney et al., 2015). Compared with a 10 min rolling average, a large effect was observed for acceleration and decelerations of 1–2 min. A moderate to small effect for 3–7 min duration was also recorded (Delaney et al., 2015). While this approach presents the maximum load of an athlete over varying durations, all acceleration and deceleration measures were modified to estimate the total number of accelerations performed (Delaney et al., 2015). This approach could be misleading as energetically, the ability to accelerate and decelerate is different. Using this approach, the specific training prescription of deceleration is consequently limited.

The deceleration output of court-based team-sport athletes remains largely unknown. Decelerations account for up to 18% of total distance covered during professional football match play (Akenhead et al., 2013). Decelerations, and their distribution over varying epochs, should therefore be included in the activity profiles of court-based team-sport athletes, to ensure appropriate training design for competition. The inconsistency previously described in defining velocity thresholds is also evident in research on decelerations. There is currently no consensus on how to define acceleration or deceleration thresholds. While presenting the acceleration frequency of team-sport athletes provides a global representation of high-intensity movements, limited research exists on the individualization of acceleration thresholds. The classification of accelerations is also dependent upon the sampling epoch utilized, which may alter the magnitude of frequencies reported.

## Filtering of data

Athlete tracking data may be filtered during the post-processing phase. Filtering involves the smoothing of position and reduction of noise using various mathematical algorithms (Carling et al., 2008). Noise can be removed by numerous techniques, each with different results. Curve fitting involves a low-order polynomial curve fitted to raw trajectory data. Although, this technique is best for repetitive movements including jumping, error may be introduced through poor selection of specific points that the curve is fitted to (Winter, 2009). These points are determined from the raw data and consequently, are influenced by the very noise the filter is trying to eliminate (Winter, 2009). Bandpass filtering converts raw data from the spatial to the time domain, typically using a Fast Fourier Transform (FFT). High-frequency signal, uncharacterize of normal human movement, is eliminated before data is converted back into the spatial domain through an inverse FFT (Wundersitz, D. et al., 2015). However, the threshold used as the optimal cut-off frequency is arbitary and typically chosen via visual inspection (Wundersitz, D. et al., 2015). Digital filtering analyzes the frequency spectrum of both signal and noise. The signal typically occupies the lower end of a frequency spectrum and overlaps with the noise, which is typically observed at a higher frequency (Winter, 2009). A low-pass filter permits the lower frequency signals while consequently reducing the higher frequency noise. Low-pass filtering can be used when analyzing trajectory data (Winter, 2009).

The filtering of athlete external load data is dependent upon the tracking system utilized. Filtering may occur on raw positional data at the instruction of the tracking system manufacturer (Stevens et al., 2014). Derived measures, including metabolic power from GPS (Di Prampero et al., 2005; Osgnach et al., 2010) are also filtered at unspecified frequencies during the post-processing stage. Butterworth (Stevens et al., 2014) and Kalman (Sathyan et al., 2012) filters are typically used for LPS data. There is limited information on how filters are used in optical player tracking systems and GPS. Filtering may account for the 24% difference in sprint distance between real-time and post-match Australian football GPS data (Aughey and Falloon, 2010) although no detail was presented on how the manufacturer explains these discrepancies. It is important to know how the manufacturer of an athlete tracking system filters raw data, particularly when inferences from external load are used to make decisions on programming training (Borresen and Lambert, 2009; Rogalski et al., 2013). The filtering of accelerometer data has recently been examined (Boyd et al., 2011). Only one of the 13 filters was strongly related (mean bias; −0.01 ± 0.27 g; CV 5.5%) to the criterion measure, Vicon (Wundersitz, D. et al., 2015). Information on filtering is rarely presented from GPS or LPS data when time spent or distance covered in velocity bands are reported. The filtering of raw data from an athlete tracking system has a substantial impact on the frequencies and distances covered in velocity or acceleration zones (Wundersitz, D. et al., 2015). Prior to reporting team-sport athlete activity profiles, researchers should detail the type of filtering applied to raw data.

## Individualized thresholds

Activity profile data reported as an average across a team (Aughey, 2011b) or position (Mooney et al., 2011; Varley and Aughey, 2013) does not account for differences in individual physical capacity. The use of a single sprinting or high-velocity threshold, for all athletes within a team, also does not consider the differences between individual athletes. Although, team-sport matches are contested at an absolute level, the same external load calculated by a high-velocity or sprinting threshold, for two athletes could represent a different internal load based on individual characteristics (Impellizzeri et al., 2004). Athlete movement may be expressed relative to a physiologically defined variable. High-intensity activity can be classified as greater than the second ventilatory threshold (VT_2_), obtained during a maximal aerobic capacity (VO_2max_) test. The VT_2_ is the point where CO_2_ production exceeds O_2_ consumption during exercise (Davis, 1985). It is assumed that activity beyond this point cannot be sustained for prolonged periods due to the athlete no longer being in a steady state (Davis, 1985). During team-sport matches, activity below the VT_2_ can likely be continued for a prolonged duration. In male soccer athletes, distance covered at or greater than vVT_2_ was 167% higher or a *very large* effect when compared to a threshold of 5.50 m·s^−1^ (Abt and Lovell, 2009). A 44% variation in athlete rank, calculated by distance covered at high-speed, was observed between the two thresholds (Abt and Lovell, 2009). Individual VT_2_ has also been measured in professional soccer athletes (Lovell and Abt, 2012). The resulting vVT_2_ was compared to an arbitrary velocity (4.00 m·s^−1^) threshold (Lovell and Abt, 2012). High-speed running distance was overestimated by 9% when arbitrary thresholds were used (Lovell and Abt, 2012). For individual athletes, this range could be between 22% lower and 33% higher (Lovell and Abt, 2012). In elite female rugby sevens athletes, a physiologically-defined threshold corresponding to treadmill speed at VT_2_ was compared to a cohort average (3.50 m·s^−1^) value (Clarke et al., 2014). When individualized thresholds were used, high-intensity running was up to 14% over or under-estimated compared to the cohort mean VT_2_ derived threshold (Clarke et al., 2014). Distance covered at high-speed may therefore be underestimated by traditional thresholds.

While the individualization of velocity thresholds is a well-reasoned approach to assess external load, conjecture exists on the implementation of an incremental treadmill protocol, conducted within a laboratory, and its application to team-sports. The individualization of velocity thresholds, derived from a continuous running protocol, does not consider the change of direction and acceleration movements, frequent in team-sports (Lovell and Abt, 2012). Whilst speed thresholds have been individualized in field-based team-sports (Abt and Lovell, 2009; Lovell and Abt, 2012; Clarke et al., 2014), limited research exists on court-based team-sports.

Athlete thresholds for external load can be expressed relative to maximum speed attained during sprint testing. The external load of junior-elite male soccer athletes was compared using absolute (>5.27 m·s^−1^) or individual thresholds by obtaining the peak running velocity during the fastest 10 m split of a 40 m sprint (Buchheit et al., 2010b). Athletes in the highest playing standard (U18 years of age) performed more repeated-sprint efforts when activity was assessed using absolute thresholds (Buchheit et al., 2010b). Younger players (U13 and U14 years of age) recorded more sprinting activity with individualized thresholds (Buchheit et al., 2010b). In junior male rugby league athletes, when an individualized threshold of peak velocity obtained during the final 20 m of a 40 m sprint test was compared with absolute speed (>5.00 m·s^−1^) thresholds, younger athletes (U13) performed *likely* (effect size = 0.43–0.58) greater high-speed running compared to their older (U14 and U15 years of age) counterparts (Gabbett, 2015). The total high-intensity running performed by junior athletes may be altered when expressed relative to a movement threshold obtained during maximal sprinting (Buchheit et al., 2010b; Gabbett, 2015). Inconsistencies therefore exist in the recorded sprinting distance according to the velocity threshold used.

Expressing a team-sport athlete's data relative to a physiologically defined threshold is an individualized approach that may benefit the training prescription for players. Although, an advancement on the use of arbitrarily derived velocity thresholds, limited research exists on how to individualize accelerations. Accelerations require more energy than constant velocity (Osgnach et al., 2010). Without information on how to classify accelerations, individualized thresholds are therefore limited in their use for team-sport athletes, including those who participate in court-based sports.

## Relationship of high-intensity activity to match performance

The capacity to accelerate and sprint is important for team-sport match performance. In junior-elite Australian Football, athletes faster over a 5 and 20 m split acquired the most kicks and disposals during matches, compared with their slower counterparts (Young and Pryor, 2007). During elite matches, a relationship exists between athlete physical capacity and the number of disposals. This relationship is mediated by the amount of high intensity-running (HIR) m·min^−1^ or distance traveled at >4.17 m·s^−1^ (Mooney et al., 2011). Sophisticated modeling techniques may therefore be able examine the effect of contextual and match-related factors on team-sport athlete running intensity.

The relationship between physical capacity and match performance in professional soccer was examined across three top English leagues (Bradley et al., 2013). Total distance covered and HIR >5.50 m·s^−1^ was captured via semi-automatic tracking (Bradley et al., 2013). Less total and HIR distance occurred at a higher than a lower playing standard. Physical capacity, defined as score on the Yo-Yo intermittent recovery two (IR2) test, was correlated with HIR distance (Bradley et al., 2013). In junior-elite male soccer athletes, the relationship between external load, defined as movement >4.47 m·s^−1^ and physical capacity, quantified as score on the Yo-Yo IR1, was position dependent. Poor correlations were observed between match running performance and athlete physical capacity in all positions except strikers. However, the 1 Hz GPS units used have poor validity (CV% of 11–30%) for assessing HIR (Coutts and Duffield, 2010). To truly quantify the relationship between athlete match external load and physical capacity, tracking technologies that are accurate at detecting movement within a range of intensities should also be used. Although, the relationship between match outcomes, athlete performance, and external load have been examined, research has applied a mean velocity threshold to all athletes within a team (Mooney et al., 2011; Bradley et al., 2013). The justification for these thresholds is typically based on other literature or arbitarily determined. Individualizing velocity thresholds may allow for a detailed analysis of the relationship between athlete external load and match outcome, although physiologically defined thresholds are limited in their application for defining accelerations (Varley and Aughey, 2013). The majority of research on the relationship between athlete performance and external load has focused on males competing in team-sports, with limited information on female athletes (Costello et al., 2014).

## Thresholds for male and female team-sport athletes

Men and women compete in team-sports at an elite level. Tracking technologies, including GPS, are used to collect the activity profiles of male and female team-sport athletes (Gabbett and Mulvey, 2008; Dwyer and Gabbett, 2012; Vescovi, 2014). There are differences in physiological capacities between sexes, including aerobic fitness and absolute sprinting ability (Mujika et al., 2009). Consequently, the physiological cost of high-speed running may be substantially different for male and female team-sport athletes. Although, lower speed thresholds are suggested for female team-sport athletes (Dwyer and Gabbett, 2012), limited research exists on the application of these thresholds. An under- or over-estimation of external load may occur if female athletes use thresholds initially developed for male athletes.

Thresholds developed for male team-sport athletes have been applied to female external load data. During international female hockey matches, the average number (17) of sprints completed was lower than the mean number (30) performed by male athletes (Macutkiewicz and Sunderland, 2011). However a sprinting threshold of 5.2 m·s^−1^, adapted from research on male soccer athletes (Bangsbo, 1992), was applied to female match data. Since there are sex differences in sprinting speed (Mujika et al., 2009), the reduction in mean sprints observed during international female hockey could be due to the inappropriate use of a velocity threshold designed for males. In soccer, male velocity thresholds have also been applied to female external load data (Krustrup et al., 2005; Mohr et al., 2008). However, the sprinting speed of female soccer athletes varies across age (Vescovi et al., 2011) and differs compared to males (Mujika et al., 2009). To develop female specific values, varying velocity thresholds have been used in soccer (Vescovi, 2012). During competitive matches, sprinting by professional female soccer athletes accounts for 5.3% of total distance covered when categorized as activity >5.0 m·s^−1^ (Vescovi, 2012). However, if the threshold is increased to >6.9 m·s^−1^, similar to thresholds used for male team-sport athletes (Varley et al., 2013b), little to no sprinting is recorded (Vescovi, 2012). A ceiling effect may therefore be present when using thresholds originally developed for male team-sport athletes. Although, the use of varying velocity thresholds is a guide in the development of sprinting values for female soccer, this approach does not consider the individual physiological differences between athletes.

The individualization of velocity thresholds for female athletes has recently been examined. In elite female rugby sevens athletes, a male velocity threshold (5.0 m·s^−1^), individual and cohort mean vVT_2_ speed, was used to determine distance covered at high-intensity (Clarke et al., 2014). The absolute amount of match high-intensity running was underestimated by up to 30% when using a velocity threshold designed for male athletes (Clarke et al., 2014). The individualized threshold under- or over-estimated high-intensity running by up to 14% when compared to the cohort mean vVT_2_ speed threshold of 3.5 m·s^−1^ (Clarke et al., 2014). Individualizing the high-intensity running threshold, assessed via a linear physiological test, of female team-sport athletes may allow for customized training prescription. However, individualization requires a time-consuming and expensive laboratory-based VO_2max_ test, which can be difficult to implement with a large number of athletes in a team-sport setting. Alternatively, the maximal aerobic speed (MAS) of an athlete is highly-correlated with maximal oxygen uptake (Léger and Boucher, 1980) and reflects running economy (Di Prampero et al., 1986). Assessment of MAS can occur on a large number of athletes during an incremental field running test (Buchheit et al., 2013). The relationship between MAS and high-intensity running has been assessed in youth male soccer athletes (Buchheit et al., 2013) although, to date, no research exists on individualizing the velocity thresholds of female team-sport athletes using MAS testing results. For female team-sport athletes who cannot complete individualized physiological or field testing, a threshold of 3.5 m·s^−1^ could be used as guide for high-intensity running, although differences between playing position and standard are not accounted for with this fixed threshold.

The development and implementation of female-specific thresholds, according to playing standard and position, should be investigated. Although, thresholds have been developed for female athletes competing in field-based sports (Dwyer and Gabbett, 2012; Clarke et al., 2014), there are no thresholds specifically for court-based sports. Netball, for example, is a court-based team-sport played indoors by elite female athletes. Due to the lack of research on female court-based sports, there is limited information on how to quantify velocity and acceleration thresholds for netball athletes.

## Alternate approaches to classify athlete activity

Data mining is a research area that aims to discover regularity from within large datasets and yield insights that are not possible using conventional statistics (Chen et al., 1996). Large databases, such as the external load obtained from tracking technologies, can therefore be investigated. Knowledge may be extracted through data mining techniques including classification, where data are sorted into predefined classes based on some common features (Chen et al., 1996). These methods are alternative approaches to the individualization of team-sport athlete external load. For example, the latent properties of external load from a single athlete can be found using data mining approaches. Velocity or acceleration thresholds are therefore derived directly from the sampled data and can be examined across age, sex, playing standard, or position.

Relationships between latent properties in data that may impact athletic performance can be uncovered using data mining (Ofoghi et al., 2013). Machine learning, a data mining technique, has been used to discover the physiological capacities required to medal in sprint cycling (Ofoghi et al., 2010). A recent review (Ofoghi et al., 2013) highlighted the lack of a contemporary framework for analyzing the match performance data of elite athletes. For example, a traditional statistical analysis on the performance of a team-sport athlete during passing chains may consider a direct relationship with a dependent variable. However, this type of analysis ignores the context of data collection (Ofoghi et al., 2013). Using data mining techniques, the hidden features that may impact upon passing quality could be examined, going beyond a superficial analysis (Ofoghi et al., 2013).

An alternative approach is mediation analysis, a statistical technique that examines the relationship between the dependent variable and independent variables to identify plus explain process. Mediation analysis has been applied in elite Australian Football to examine inter-relationships between athlete capacity, match intensity and performance (Mooney et al., 2011). Playing position and experience influence the relationship between an athlete's capacity, match activity profile and possession output (Mooney et al., 2011). Linear techniques including discriminant analysis (Castellano et al., 2012) and generalized linear modeling have also been used to examine team-sport performance. However, linear techniques may not be an optimum method to analyze the match performance of dynamic and chaotic team-sports.

In contrast, non-linear data mining techniques are not constrained to a single linear variable. Decision trees, a non-linear technique, have been used to explain match outcome in Australian football (Robertson et al., 2016), classify team-sport activities from a wearable sensor (Wundersitz, D.W. et al., 2015) and explore the attacker and defender interaction during invasion sports (Morgan et al., 2013). Decision trees involve the repeated partitioning of data, based on input fields that create branches which can be further split to differentiate the dependent variable. Decision trees can handle missing data and provide an intuitive analysis of a dataset (Morgan et al., 2013). Unlike clustering, decision tree induction is not dependent on the selection of a prior distribution.

Clustering is a data mining technique that could be used to find unknown patterns in large datasets by classification, whereby data is grouped based on similarity (Chen et al., 1996). A large dataset can be meaningfully divided into smaller components or categories using clustering (Punj and Stewart, 1983). These categories may be mutually exclusive (Fayyad et al., 1996). Categories can also be sorted in a hierarchical or overlapping manner. Gaussian mixture models, a cluster method that contains a prior belief about group assignment, have been used to classify shot making in tennis (Wei et al., 2013). These clustering methods represent sub-populations within a dataset and express the uncertainty about cluster assignment. The *k*-means clustering algorithm divides a dataset into a user-specified number of *k* clusters (Wu et al., 2008). The *k*-means algorithm starts with *k* centroids, selected at random. Each data point within the wider dataset is assigned to its nearest centroid, based on similarity. The centroids are updated each time a data point is assigned (Wu et al., 2008). The centroid mean is then calculated from the data points allocated to that cluster (Wu et al., 2008). The size of the dataset determines the number of repetitions required for the *k*-means algorithm to reach completion (Wu et al., 2008). Clustering, via the *k*-means algorithm, could be used in a variety of sport settings, including grouping the external load of an athlete.

Complex statistical or data mining techniques, including clustering, may uncover unknown patterns or counter prior beliefs. These approaches could be used to guide the development of athlete velocity and acceleration thresholds. Self-organizing maps (SOM) and clustering have been utilized in elite rugby union to uncover playing styles related to team success (Croft et al., 2015). The coordination patterns during three different basketball shots from varying distances have also been classified using SOM (Lamb et al., 2010). The lowest variability was recorded in the three-point and hook shots. The SOM displayed a movement output that differed unexpectedly from traditional analysis, including visual inspection and time series data (Lamb et al., 2010). A movement analyst with experience and prior knowledge or bias may have been distracted by other information compared to a SOM, that has a more objective methodology (Lamb et al., 2010). These approaches could also be used to group athlete velocity data, without the requirement of a human input threshold based on a physiologically defined or arbitary value. These groups could be formed irrespective of an athlete's age, sex, position, or playing standard. Patterns within athlete movement, including velocities and accelerations performed, could be derived by applying clustering techniques to external load data.

The accelerometer derived PlayerLoad™ data of elite female netball athletes was grouped by *k*-means clustering (Young et al., 2016). Optimal clustering was the greatest Euclidean distance obtained from two to five clusters (Young et al., 2016). The seven netball playing positions were divided into two groups according to playing intensity and relative time spent in a low-intensity zone (Young et al., 2016). The PlayerLoad™ for the goal based positions was lower than the attacking and wing positions, likely due to the time spent performing low intensity activity (Young et al., 2016). This study was the first to use data mining techniques, including *k*-means clustering, to examine athlete load data. However, only accelerometer data was investigated and not the position of an athlete, from GPS or LPS. Capturing the position of an athlete allows for the calculation of displacement, velocity and acceleration. With the large volume of data obtained from athlete tracking systems, data mining represents a technique to gain further insight into athlete activity profiles. Consequently, athlete external load could be analyzed without the requirement of an arbitrary or software-implemented threshold.

## Recommendations

A range of velocity thresholds are utilized to classify the sprint effort of a team-sport athlete. Although, thresholds may be individualized (Abt and Lovell, 2009; Clarke et al., 2014), applying a global velocity or acceleration threshold may allow for examination of positional and individual differences over time. A practical issue for those monitoring activity profiles is determining velocity and acceleration thresholds for a cohort of athletes. Selection of these global thresholds is often arbitary and dependent upon the cohort profiled. We recommend that practitioners choose thresholds of an equal bandwidth, for example, 0–5, 15–10, 15–20, 20–25, and ≥25 km·h. The minimum duration required for a sprint effort to be recorded should also be stated.

For elite female team-sport athletes competing in field-based sports, a fixed threshold of 3.5 m·s^−1^ may be used to detect high-speed activity across a cohort of players (Clarke et al., 2014). Since a consensus is yet to be reached on the physiological tests to determine velocity or acceleration thresholds, we recommend that practitioners chose a test deemed most appropriate for their sport. Alternatively, data mining approaches could be used to examine the velocity and acceleration output of team-sport athletes. Recently, the velocity, acceleration and angular velocity output of court-based team-sport athletes was examined without arbitary thresholds (Sweeting et al., 2017). Rather than comparing the velocity, acceleration and angular velocities performed by individuals as a function of time, the similarities between playing positions according to the movement sequences performed. This approach may have application for coaching and conditioning. Knowledge of the movements performed, angle of attack and accelerations may assist with planning sport-specific training. Practitioners and scientists can subsequently focus on training the specific movement sequences frequently performed by athletes in each playing position. These sequences can also be examined across different playing standards, such as elite and junior-elite levels. Profiling the activity profile across the athlete pathway may assist in preparing team-sport athletes during transition from lower to higher levels.

## Conclusion

Athlete position, velocity, and acceleration can be measured during matches or training via optical tracking, GPS and LPS. The analysis of distance, velocity, and acceleration over a specified time epoch is termed athlete activity profile. It is difficult to compare literature on field-based sports due to inconsistencies in velocity and acceleration thresholds, even within a single sport. Velocity and acceleration thresholds have been determined from physiological and physical capacity tests. Limited research also exists on female team-sport athletes and how to classify their velocity plus acceleration. Alternatively, data mining can derive patterns from large datasets. With the large volume of data obtained from athlete tracking systems and advancements in classifying movement patterns during skill or endurance performance, data mining is a technique to gain further insight into athlete activity profiles. Consequently, athlete external load could be analyzed without velocity or acceleration thresholds. Future work should focus on using data mining techniques to analyze the movement performed by team-sport athletes, particularly elite females and those participating in court-based sports.

## Author contributions

Conceived and designed the experiments: AS, SC, SM, and RA. Drafted manuscript and prepared tables/figures: AS. Edited, critically revised paper, and approved final version of manuscript: AS, SC, SM, and RA.

### Conflict of interest statement

The authors declare that the research was conducted in the absence of any commercial or financial relationships that could be construed as a potential conflict of interest.
